# An Overview of Supramolecular Platforms Boosting Drug Delivery

**DOI:** 10.1155/2023/8608428

**Published:** 2023-11-13

**Authors:** Rosa Bellavita, Simone Braccia, Annarita Falanga, Stefania Galdiero

**Affiliations:** ^1^Department of Pharmacy, University of Naples ‘Federico II', Naples 80131, Italy; ^2^Department of Agricultural Sciences, University of Naples ‘Federico II', Portici 80055, Italy

## Abstract

Numerous supramolecular platforms inspired by natural self-assembly are exploited as drug delivery systems. The spontaneous arrangement of single building blocks into inorganic and organic structures is determined and controlled by noncovalent forces such as electrostatic interactions, *π*-*π* interactions, hydrogen bonds, and van der Waals interactions. This review describes the main structures and characteristics of several building blocks used to obtain stable, self-assembling nanostructures tailored for numerous biological applications. Owing to their versatility, biocompatibility, and controllability, these nanostructures find application in diverse fields ranging from drug/gene delivery, theranostics, tissue engineering, and nanoelectronics. Herein, we described the different approaches used to design and functionalize these nanomaterials to obtain selective drug delivery in a specific disease. In particular, the review highlights the efficiency of these supramolecular structures in applications related to infectious diseases and cancer.

## 1. Introduction

**Figure 1:**
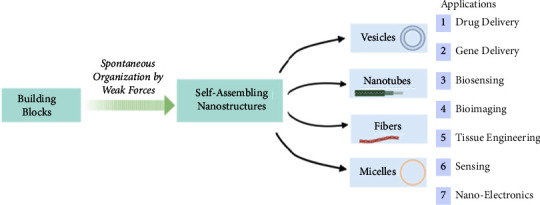
Representation of some supramolecular nanostructures and their biological applications. This figure was created with https://BioRender.com.

Motivated by the extensive presence of self-assembly in nature, researchers have explored the self-assembly process of natural macromolecules to push chemical self-assembly and develop a series of advanced designs with defined structure, size, function, and application [1, 2]. Furthermore, since natural systems rarely occur as isolated structures but are more often the result of interacting assemblies, nanoscience is slowly moving from the development of nanomaterials with fascinating structures to their assembly into larger architectures [3]. These supramolecular assemblies find application in diverse fields ranging from drug/gene delivery, theranostics, catalysis, tissue engineering, and nanoelectronics (see Figure 1) [4, 5]. Self-assembly is a valuable and straightforward strategy to produce complex functional materials, as witnessed by the remarkable advancements in this field with a number of newly designed synthetic systems being developed [6]. At the heart of each function is a carefully designed nanosystem, where the relative arrangement of building blocks dictates their final optical and physical properties along with the distinct morphology of the obtained self-assembled nanostructures such as fibers, nanotubes, vesicles, and micelles [7].

Self-assembly is the natural growth of ordered structures with no assistance from an outer agent [8], determined by forces (such as electrostatic interactions, *π*-*π* interactions, hydrogen bonds, van der Waals interactions, and hydrophobic interactions) that are individually very weak (2–250 kJ/mol) compared to covalent bonds (100–400 kJ/mol) [9], but together are sufficiently strong to produce stable self-assembled functional materials [10]. The double-stranded DNA, the triple helix of collagen, the structure of viruses, and the membrane bilayers are just a few examples of self-assembly in nature. The magnitude and type of interactions influence the shape, function, and size of the final assembled nanomaterial [11]. Self-assembled materials can be static if they do not change with time and there is no exchange of energy with the environment such as molecular crystals and globular proteins, or dynamic if they are in a nonequilibrium state with a constant dissipation of energy into the environment such as living cells [12]. To investigate the structure-assembly relationship and develop new architectures, various building blocks such as amphiphiles, peptides, dendrimers, polymers, fullerenes, calixarenes, cyclodextrins, and lipids were designed and synthesized [13]. In addition, the ability to understand, regulate, and control the pathways and the final thermodynamic state that govern the complex interactions between the individual components is essential [14], and to establish a collection of next-generation materials, it is fundamental to have a thorough comprehension of the design principles to predict the outcome of the self-assembly process [15].

This review aims to focus on the powerful role of finely tuning self-assembly to succeed in obtaining the preferred structures with the desired properties. As bridging the design and prediction of the self-assembly process will certainly have a great influence in medicine; here, a description of the designed self-assembly process is delivered with a report on the structural features and possible applications in infectious diseases and cancer [16].

The review is mainly divided into five parts: (i). self-assembling strategies; (ii). self-assembled nanosystems and corresponding assembly morphology (liposomes, LNP, self-assembled peptides, etc.); (iii). the recent advances in applications for infectious diseases; (iv). the recent advances in applications for cancer; and (v). biophysical techniques to characterize self-assembled nanosystems.

## 2. Self-Assembling Strategies

Self-assembly is a key theme in contemporary science with great attention given to the identification of the driving forces that regulate this process and to the supramolecular nanostructures obtained and to the physical, chemical, and biological properties that can be attained [17]. The collective behaviour of the self-assembled nanostructures may determine advanced functions and additional properties compared to their building blocks and may enable further tasks by inclusion of more functional molecules [12]. Supramolecular materials can be attained through a bottom-up strategy from a variety of building blocks, such as atoms, small molecules, or macromolecules [10]. Furthermore, modifying the chemical composition, the length, and the directionality of interactions of existing building blocks allows the design of new units containing the necessary features for their self-assembly. The spontaneous formation of self-assembled structures is a situation of minimum energy achievable by controlling environmental variables; as the driving forces are noncovalent interactions, self-assembly is a reversible process sensitive to the environment and on-demand properties might be obtained by tuning the self-assembly [18].

**Figure 2:**
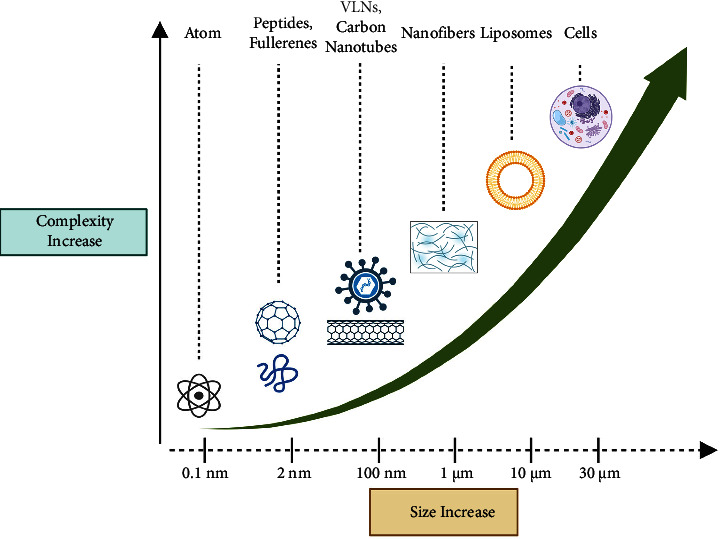
Representation of the molecular complexity of self-assembled nanostructures in the function of the increase of their size. This figure was created with https://BioRender.com.

Several morphologies can be obtained according to the building blocks used, for instance, vesicles, micelles, ribbons, helices, spherical nanoparticles, nanorods, nanotubes, and nanofibers, whose complexity increases as a function of their size (see Figure 2).

Micelles are supramolecular aggregates of polymeric amphipathic molecules approximately spherical in shape [19, 20], which in water form a structure with the hydrophilic head regions interacting with the hydrophilic aqueous environment, while sequestering the hydrophobic domains in the micelle core. Inverse micelles have the head groups at the core with the tails extending into the water solution.

Vesicles are self-contained structures consisting of an aqueous compartment surrounded and enclosed by a hydrophobic layer. The tendency of hydrophobic molecules to clump together in an aqueous environment can lead to the spontaneous formation of vesicles [21]. Thus, vesicles are made up of molecules containing hydrophilic heads and hydrophobic tails that cluster together. If there is only one layer, the vesicles are called unilamellar; otherwise, they are called multilamellar [22].

Spherical nanoparticles comprise a filled-core material [23]. These kinds of nanoparticles include a huge variety of nanosystems which may be made of one or two components; usually, there is a densely packed shell around a core nanoparticle [24].

Nanotubes are structures forming tubes with an interior hollow space; usually, these cylindrical structures have diameters of ∼1–100 nm [25]. Typical biological examples of nanotubes are transmembrane ion channels. Nanotubes can be obtained from carbon, silicon, boron, carbohydrates, polymers, and peptides. Nanofibers are long thread-like structures; their diameter is less than 100 nm, and they do not have the internal cavity, which clearly differentiates them from nanotubes [26].

## 3. Self-Assembled Nanosystems

**Figure 3:**
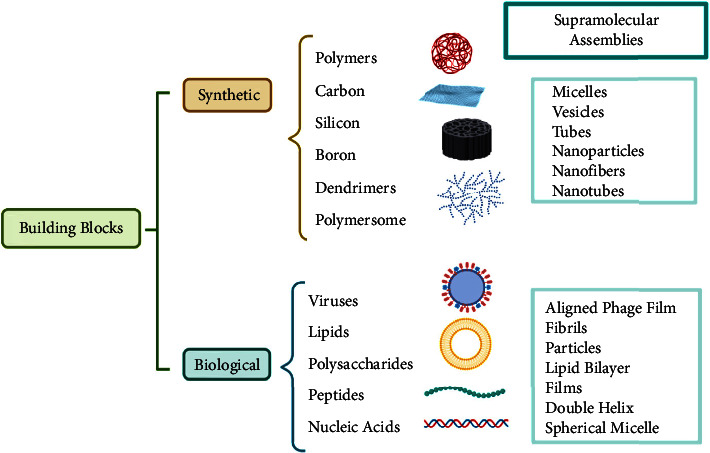
The overview of building blocks used for obtaining several self-assembled nanostructures. This figure was created with https://BioRender.com.

Building blocks for engineering a multiplicity of architectures with distinct morphologies can be achieved from a wide assortment of molecules through noncovalent interactions in each solvent. The main synthetic and biological building blocks are lipids, peptides, dendrimers, cyclodextrins, viruses, nucleic acids, polysaccharides, etc., and according to the building block, different supramolecular assemblies may be obtained, as reported in Figure 3. Essentially, the building blocks can be atoms, small molecules, or macromolecules, and they can be designed to comprise all the necessary instructions that govern their self-assembly for each specific function. The weak chemical interactions provide a high degree of directionality to the assembly process and are exploited in the design process. Furthermore, those interactions can be triggered by environmental variables, which can be controlled to push the system toward a specific self-assembled structure. Specifically, the assembly can be influenced by different extrinsic factors, including thermal treatment, chemical reactions, and mechanical stress, which establish the morphology of self-assembled nanostructures [27].

### 3.1. Lipids

Lipids are composed of a hydrophilic head group and a hydrophobic tail region and represent key building blocks [28]. In fact, thanks to their hydrophobic nature and rigid structure, they tend to aggregate into larger structures. Liposomes are self-assembled spherical vesicle with the diameter ranging from 10 to 1000 nm; a central aqueous core is enclosed by a phospholipid bilayer. Depending on the size, liposomes can be further divided into small unilamellar (SUVs: 20–100 nm), large unilamellar (LUVs: 100–500 nm), and multilamellar (MLVs: 500–5000 nm). In addition, the different nature of lipids characterized by distinctive polarities, charged groups, and lengths tolerates the establishment of more or less dynamic structures in water (*such as* monolayers, bilayers, micelles, and vesicles). The amphiphilic nature of liposomes can be exploited to encapsulate both hydrophilic and hydrophobic drugs [29]. A particular type of lipid vesicle is represented by lipid nanoparticles (LNPs), which contain a homogeneous lipid core. LNPs are widely used for the delivery of peptides, small-molecule drugs, and nucleic acids and have recently gained much attention because of their successful application as a delivery platform for COVID-19 mRNA vaccines [30–32]. LNP for gene delivery are based on the spontaneous aggregation of positively charged lipid components into nanostructured entities and have also established thanks to their electrostatic interaction with negatively charged nucleic acids [33].

### 3.2. Dendrimers

Dendrimers are synthetic, well-defined, and monodispersed molecules which arrange with a symmetrically multibranched core-shell architecture and may also assemble, through noncovalent interactions, into more complex structures (nanoparticles and nanofibers) [34, 35]. Dendrimers can be polyanionic and polycationic according to their terminal groups; for polyanionic dendrimers, there are terminal groups such as sodium carboxylate, hydroxyl, and succinamic acid, whereas for polycationic dendrimers, we likely find primary amines as terminal groups [36]. Divergent or convergent branching methods may be used for their stepwise synthesis. Many different dendrimers were recently developed, including polyamidoamine (PAMAM), poly (propylene imine) (PPI), poly (glycerol-co-succinic acid), poly (L-lysine) (PLL), poly (glycerol), poly (2,2-bis (hydroxymethyl) propionic acid), and melamine [36]. Interestingly, polyester dendrimers are biodegradable with hydrolysis rates differing dramatically according to the hydrophobicity of the monomer, steric environment, and the behaviour of functional groups within the dendrimers, while polyamine and polyamide dendrimers, show poor degradability which highly limits the range of applications *in vivo,* as they cause accumulation because of long-term therapies. Dendrimers represent a challenging strategy for the simultaneous delivery of multiple therapeutics to target sites and their inner compartment can be used to accommodate a cargo.

### 3.3. Hydrogel

Hydrogels are three-dimensional polymeric porous structures formed by physical or chemical crosslinking and able to house various materials, including small molecules, polymers, and particles and with high water absorption capacities (>90%). Physical cross-linking strategies include hydrophobic interactions, hydrogen bonding, polymerization entanglement, *π*–*π* stacking, etc. and typically confer poor mechanical strength, while covalent cross-linking including free radical polymerization, enzyme-induced cross-linking, etc., confers high mechanical properties [37]. Hydrogel is obtained by either natural (*such as* chitosan, gelatine, and polynucleotides) or synthetic polymers (such as poly (vinyl alcohol), poly (ethylene glycol), and poly (sulphobetaine)) [38]. Their properties are also determined by the material composition/concentration and fabrication approaches. The cross-linked network can hinder penetration of some proteins and can also protect loaded bioactive therapeutics (for example, recombinant proteins, antibodies, and peptides) from premature degradation, making hydrogels appealing nanomaterials for the delivery of a large variety of therapeutics. For instance, Bahmanpour et al. have proposed a novel hydrogel formulation consisting of chitosan, chitosan quaternary ammonium salt, and gelatin for intranasal insulin administration [39]. As promising materials, they present several features such as biocompatibility, predictable degradation rates, tunable mechanical properties, and good elasticity [40]. A key parameter for drug delivery applications is represented by drug loading and drug release. As hydrogels swell in the presence of water, most of them result in fast drug release that is not so useful for practical applications, especially when the desired duration of drug delivery needs to be longer.

### 3.4. Peptides

Amino acids constitute peptides and proteins, and their side chain properties (charged, polar, nonpolar, aliphatic, and aromatic) are responsible of important noncovalent interactions which makes them simple building blocks to exploit for fabricating complex supramolecular assemblies [41]. Furthermore, peptides, obtained by the coupling of a series of amino acids, constitute versatile assembly units owing to their sequence intrinsic functional diversity and are broadly used for the obtainment of self-assembled systems with unique properties [42, 43]. As self-assembling building blocks, peptides are relatively easy to prepare by solid-phase synthesis, providing plenty of opportunities to unravel the effects of molecular design on their properties and functions. The intrinsic high biocompatibility, high biodegradability, and low immunogenicity are additional beneficial properties that have always fascinated scientists to develop functional nanomaterials made of peptides. Their self-assembly is spontaneous and reproducible and by varying systematically, the sequence length and the nature of the amino acid side groups, a variety of diverse nanostructures can be produced. Furthermore, it is possible to incorporate nonpeptide groups (such as aromatic, aliphatic, and organometallic moieties) to obtain pseudopeptides characterized by extra functions such as molecular rigidity and biological activity. Peptides and pseudopeptides can be used to establish diverse supramolecular designs, for instance, vesicles, helices, nanotubes, sheets, nanorods, nanofibers, nanospheres, nanotapes, etc., and up to now, scientists have explored only limited architectures out of the large available chemical space. Clearly, a crucial property that can be tuned for peptides is their amphipathic nature. Peptide sequences with high assembling ability might be developed through alkylation or lipidation [43]. Self-assembling properties can be granted to peptides through several strategies including the binding of a hydrophobic tail to a hydrophilic peptide, which augments peptide hydrophobicity, smoothing its self-organizing ability. Micelles or bilayer structures are dependent on peptide concentrations; in fact, nanofibers or nanotubes can be obtained at higher concentrations and can also further self-assemble to form three-dimensional hydrogels [18]. Furthermore, pH and ionic strength are key features to exploit for on-demand control of peptide assembly and disassembly. Self-assembling peptides have attracted applications in many areas including drug delivery, tissue engineering, gene delivery, antibacterial agents, theranostics, vaccine development, catalysis, and electronics. For instance, drug delivery involves drug loading into nanocarriers either by encapsulation or conjugation and the controlled release of the drug at target sites. These tasks can be accomplished by incorporating appropriate functionalities such as multiple cell-targeting peptide sequences and cell-penetrating peptides (CPPs) [44].

### 3.5. Virus-Like Nanoparticles (VLPs)

Viruses-, protein-, and nucleic acid-based supramolecules are interesting building blocks thanks to their shape and size uniformity and their natural functionality through self-assembly [45]. Normally, the outer shell of a virus possesses several copies of proteins arranged in a symmetrical fashion, which contain the viral genome. Thus, the capsid self-assembly is a spontaneous process between protein monomers, representing the building blocks [46]. During the nucleation and growth phases, weak interactions are responsible for the self-association which leads to different structural arrangements, such as helical, icosahedral, spherical, or more complex shapes. Thanks to their higher biocompatibility, capacity for cell internalization, and ease of functionalization for cell targeting through genetic or chemical manipulation of their surface properties without disrupting their integrity and morphology, VLPs are highly convenient units for self-assembling [37]. Thus, VLPs are self-assembled nanoparticles, commercially approved by the US Food and Drug Administration (FDA) since the 1980s [37, 46]. Their properties, such as heterogeneity, and highly ordered structural organization are widely used to develop monodisperse nanocarriers (range size 20–500 nm) for several applications such as drug and enzyme delivery and gene therapy. Furthermore, VLPs were broadly exploited to develop vaccines against several pathogenic viruses and diverse types of cancer. Current efforts in the study of VLPs are dedicated to unravelling their capsid self-assembly mechanisms to improve their potency, efficacy, and cargo loading capacity.

### 3.6. Polymersome

Polymersomes are self-assembled polymers made of synthetic block amphiphiles [47], which although being amphipathic similarly to lipids, have higher molecular weights and in fact may be appropriately compared to viral capsids. The choice of synthetic polymers and their molecular weight are key parameters that confer on polymersomes distinctive molecular features and specific carrier properties. The polymers used to design and develop polymersomes, similarly to lipids, are amphiphiles made of a hydrophilic fraction and a hydrophobic fraction. When there is a suitable proportion of amphiphilicity, the hydrophobic motifs of each polymer self-assemble with each other to reduce the exposure to water, while the hydrophilic motif is exposed towards face inner and outer hydrating solutions. Several polymers forming polymersomes are commercially available, for example, polyethylene glycol-polylactic acid (PEG-PLA) and PEG-polycaprolactone (PEG-PCL).

### 3.7. Nucleic Acids

Individual nucleic acids such DNAs and RNAs can assemble in a controlled fashion by hydrogen bonds and *π*-*π* stacking interactions, forming a variety of nanostructures featuring different compositions, geometries, and functionalities [48]. RNA tertiary motifs can be used to form nanostructures, where all individual RNA building blocks can self-assemble in a particular way to have the specific geometry [49]. Another powerful strategy is called DNA origami and it exploits longer ssDNAs stapled by shorter oligos for the controlled formation of DNA nanostructures [50]. Nucleic acid nanoparticles have several benefits, such as the possibility to control and have the particular size, charge, and composition, as well as stability under a variety of external conditions (e.g., radiation and temperature), since they are usually degraded by traditional oligonucleotides. In addition, the nucleic acid-based technology is considered highly versatile and allows for the modification of individual nucleotides without altering the entire nanoparticle formation [51].

### 3.8. Peptide Nucleic Acid (PNA) Molecules

The self-assembly of PNA has recently been attracting attention for its many potential applications. PNA-based nanostructures originate from the combination and assembly of nucleic acids and peptide with specific properties [52]. Swenson et al. [52] explored the role of the amino acid backbone on the assembly, placing hydrophobic or hydrophilic side chains in tactical positions to engineer amphiphilic structures and control their self-assembly. Besides, the addition of complementary DNA/RNA strands determined the formation of duplexes, affecting amphiphilicity and favouring the formation of micellar structures in water solutions. They also demonstrated stimuli-responsive disassembly of the supramolecular structure using miRNA-21, a disease-related RNA target [53]. For example, the Stupp group described the development of a responsive PNA-based hydrogel [54]. The PNA self-assembled into fibers, crosslinked with double helix formation by interactions with complementary base pairs, and influenced the physicochemical properties of the gels. Clearly, to enrich and fine-tune peptide-based hydrogel properties, researchers work on PNA modifications [55]. Remarkably, hybrid materials were developed by the conjugation of PNAs to porphyrin or boron-dipyrromethene [56], which readily self-assembled in solution, forming nanoparticles with uniform particle sizes. These PNA-based nanoparticles efficiently interacted and internalized into cancer cells, providing promising results for the rational design of photodynamic nanoagents for cancer therapy.

### 3.9. Polysaccharides

Natural polysaccharides present several advantages, including good biocompatibility and low immunogenicity and toxicity, making them appropriate for engineering materials with biomedical applications. Polysaccharides have significant chemical and structural diversity (e.g., linear or branched, random coil, or helical conformation) and present several functional groups, such as OH, –COOH, and –NH_2_, that are used to engineer polysaccharides-based nanoparticles (NPs) [57]. Amphiphilic carbohydrates including hyaluronic acid and chitosan are the most used polysaccharides to build NPs [58]. These polysaccharides are made of a carbohydrate as a hydrophilic block, which is covalently attached to the hydrophobic block. Generally, when these carbohydrates self-assemble in solution, they form NPs characterized by an outer-layer hydrophilic shell and a hydrophobic inner core which is considered a reservoir to encapsulate imaging and therapeutic agents. In addition, the hydrophobic building-block controls the self-assembly and defines size, surface potential, and morphology of nanovectors. Among the several polysaccharides, chitosan (CS) made of *β*-1,4-linked N-acetyl-2 amino-2-deoxy-D-glucose and 2-amino-2-deoxy-D-glucose units [59], is the most favourite block for engineering biological materials since it presents excellent biodegradability, biocompatibility, and low toxicity. CS possesses functional groups such amino and hydroxyl groups that are exploited for several applications [60]. For instance, the presence of amino groups in CS that are able to chelate metals makes CS suitable for removing heavy metals during wastewater treatment, while the hydroxyl group can be involved in etherification and esterification reactions that are used for the CS functionalization and the formation of biomedical nanomaterials. Hyaluronic acid (HA), composed of alternating D-glucuronic acid (GlcUA) and N-acetylglucosamine (GlcNAc) blocks, is a further polysaccharide widely used for manufacturing delivery systems in the biomedical field [61]. Being biocompatible and biodegradable, HA plays many roles in tissue function and development, cosmetics, and disease treatment [62]. HA-based nanostructures are divided into organic and inorganic nanomaterials. Examples of HA-based organic nanomaterials are micelles obtained through the self-assembly of HA conjugated to hydrophobic molecules. In the micelle's core, the hydrophobic drugs can be encapsulated via chemical conjugation, physical absorption, or electrostatic interactions. The organic nanomaterials also include nanogels obtained through conjugation of HA with the hydrophobic group, such as methacrylate group or cholesterol. These latter have high drug-loading efficiency and high colloidal stability to be deemed safe drug delivery systems. HA-based inorganic nanomaterials have also aroused interest in the physicochemical properties of inorganic materials, including inertness, stability, and easy functionalization [63]. Examples are silica nanoparticles (SiNPs) and gold nanoparticles (AuNPs) functionalized with HA and used in chemotherapy, photothermal therapy (PTT), photodynamic therapy (PDT), and diagnostics. In these cases, HA is used for targeting cancer cells because it can bind overexpressed receptors such as CD44 and RHAMM on cancer cells. Moreover, among the polysaccharides, cyclodextrins are widely exploited for engineering drug delivery systems. CDs are made of different D-glucopyranoside units linked through an *α*-linkage; the most abundant natural CDs consist of six (*α*CD), seven (*β*CD), and eight units (*γ*CD). CDs assume the structure of a truncated cone having a hydrophilic exterior and a hydrophobic interior, which favours the formation of inclusion complex where a lipophilic molecule is entrapped in the hydrophobic CD cavity, while a hydrophilic molecule can interact on its surface. Thus, in the pharmaceutical field, CDs are generally used to enhance solubility and bioavailability of hydrophobic drugs. For instance, in water solution, *γ*CD, thanks to the hydrogen bonding driving force of the external OH groups, can self-assemble to form aggregates in a concentration-dependent manner [64]. This is a dynamic equilibrium, where *γ*CD aggregates are consistently formed and dissociated in aqueous solutions. The self-assembled *γ*CD may aid in increasing the solubility and enhancing drug permeability through biological membranes.

### 3.10. Graphene Oxide

Graphene oxides have attracted great attention as carriers for drug delivery because of their exceptional biocompatibility. Furthermore, graphene oxides present tremendous flexibility which allows them to assemble into different shapes such as spherical microcapsules which have a coreshell structure that can effectively load diverse drugs [65]. Machado and collaborators reported on nanosheets of graphene oxide assembled with layers of a biodegradable polymer between the drug-containing monolayers to achieve a time-controlled drug delivery system [66]. An increase in the number of graphene oxide layers corresponded to a proportional release delay and, in particular, the kinetic release was tuned as a function of the number of layers [66]. Another interesting paper reported about graphene oxide nanosheets loaded with magnetic iron oxide nanoparticles for the obtainment of chitosan/sodium alginate functionalized nanocomposites with application in targeted cancer drug delivery and photothermal therapy [67].

## 4. Self-Assembly by Preassembled Nanoassemblies

A new attractive frontier in the supramolecular chemistry is represented by the self-assembly of preassembled nanoassemblies. This challenging approach allows the construction of multilevel architectures that are highly complicated, featuring high versatility and a wide range of applications. Several kinds of copolymer-preassembled nanoassemblies might be exploited as subunits for higher level self-assembly. Apart from micelles or vesicles, to develop self-assembly from preassembled nanoassemblies, it is necessary to introduce interactions among subunits, which include electrostatic interactions, crystallization-driven contacts, and other kinds of intermolecular forces.

**Figure 4:**
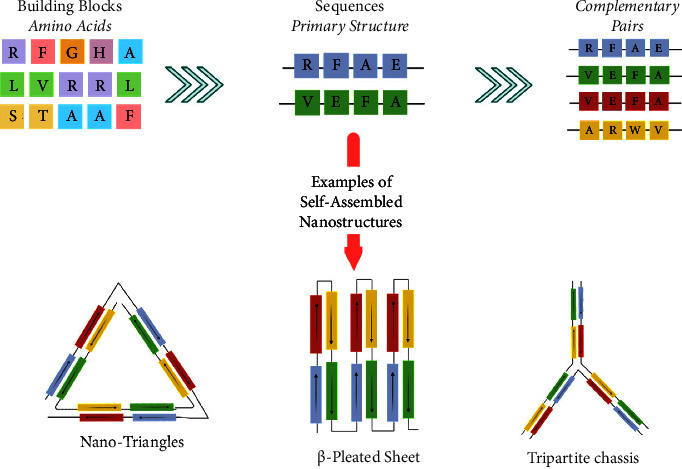
Schematic representation of short peptide motifs containing complementary pairs that induce the self-assembling into nanostructures. This figure was created with https://BioRender.com.

DNA origami nanostructures and supramolecular DNA-based structures possessing self-complementarity present many applications in material science and nanomedicine. The DNA origami technology constitutes a versatile approach in assembling two- and three-dimensional structures of nucleic acid constituents [50]. The use of DNA strands as scaffolds yields diverse DNA “origami” nanostructures and establishes a mean to functionalize the origami tiles to achieve a desired activity [68, 69]. Furthermore, substantial research efforts are dedicated to the engineering of 3D origami which self-assembles into supramolecular nanostructures exhibiting exiting properties [57, 70–72]. On the basis of the principle of DNA origami, the design of complex nanostructures characterized from tens to hundreds of peptide chains origami has not been reported yet [73]. The single amino acid residues are not able to form with each other a strong conjugation like that of base pairs unlike short peptide motifs which are more suitable to realize the orthogonal complementary pairing and form the origami (see Figure 4). Interestingly, peptide motifs present several advantages over DNA base pairs, thanks to the numerous and strong interactions occurring between the amino acids. Among peptide motifs, *β*-sheets and coiled-coils motifs derived from some natural proteins such as insulin and *α*-synuclein showed the capability to self-assemble forming origami. The choice of the kind of protein and correlated peptide motifs can be performed by using specific software.

Anyway, although the fabrication of origami by exploiting peptide self-assembly remains still a hard challenge, different studies were performed. For instance, Fletcher and co-workers used two coiled-coil motifs that self-assemble into hollow balls with a diameter of 100 nm forming a single-layered protein grid [74]. Similarly, Gradišar and co-workers have constructed a complex tetrahedral structure featuring a 10 nm length by assembling six coiled-coil orthogonal complementary pairs [75]. Even more attractive is the fabrication of artificial viruses performed by Noble and co-workers [76]. In this work, the authors used a coiled-coil peptide helix which self-assemblies into anionic virus-like shells in which RNA and DNA can be encapsulated and transferred in human cells.

Hence, as described above, thanks to their feasibility and superiority, peptide motifs have a significant potential to engineer hybrid origami structures with a wide range of biomedical applications.

## 5. Applications for Infectious Diseases

Drug resistance represents a great uncertainty in current medicine, and new strategies to address infectious diseases are needed without delay to avoid widespread susceptibility against infections that so far have been straightforwardly treated with existing traditional antibiotics/drugs [73, 77]. One promising field of research investigated to address the phenomenon of antibiotic resistance consists of the use of self-assembling nanomaterials for their ability to deliver and target existing antimicrobial agents against the microbial targets [44]. Dendrimers have shown great promise as delivery vehicles; cationic dendrimers possess great promise as selective antibacterial; the most active are poly(amidoamine) (PAMAM) dendrimers carrying several charged terminals which unfortunately are associated with significant cytotoxicity at their MIC concentrations [78].

Dhumal et al. have demonstrated that an amphiphilic dendrimer made of a long hydrophobic alkyl chain and a small hydrophilic PAMAM dendron carrying distinct terminal functionalities interacts and binds with the bacterial membrane by forming electrostatic interactions; through self-assembly into supramolecular structures, they destroy the bacterial membrane [79].

We also reported the efficient use of bifunctional peptidodendrimer orthogonally conjugated with two different antiviral peptides derived from the glycoproteins gH and gB of herpes simplex virus type 1 (HSV-1). The resulting bioconjugate demonstrated no toxicity to cells at biologically relevant concentrations and good inhibition of both the early and the late stages of the herpes infection process [80]. Tarallo and co-authors reported a PAMAM dendrimer functionalized with a CPP, namely, gH625, which inhibits HSV-1 (half-maximal inhibitory concentration, IC_50_ was 100 nM) and HSV-2 (IC_50_ was 300 nM) at a very early stage of the entry process of infection, preventing the virus from interacting with cellular membranes, and no evidence of cell toxicity at these concentrations was reported [81].

Moreover, the internal cavity of dendrimers is favourable to encapsulate a huge variety of hydrophobic antiviral drugs, i.e., ritonavir and efavirenz. Pyreddy and co-workers synthesized a fifth-generation efavirenz (EFV) conjugating PEGylated PAMAM dendrimers through an ethylenediamine central core by using Michael addition [36, 82].

Antibiotics have also been coupled to dendrimers to enhance their antimicrobial activity, half-life, and solubility [83, 84]. Penicillin when bound to a PAMAM dendrimer through a PEG linker, retained its activity and was effective against *Staphilococcus aureus.* In particular, the drug was released only when in close proximity to the bacteria [85]. Similarly, dendrimer-based vancomycin conjugates were more active against vancomycin-resistant and nonresistant *S. aureus* with nanomolar affinity [86]. In addition, dendrimers are smart platforms that can be functionalized with more molecules acting in different manner. For example, Fernandez et al. described the strong combination effect of levofloxacin and a cation carbosilane dendron alone and conjugated to the cell-penetrating peptide gH625 (developed previously by some of us) to treat *S. aureus* biofilms. When the dendron is conjugated to gH625, its efficiency is increased, and the effect on biofilm viability was observed at the lower amount of dendron than the dendron alone [87].

Supramolecular assembly of peptides has been widely exploited as an effective method to obtain AMPs with increased stability, antimicrobial efficacy, decreased haemolytic response, and enhanced stability to serum proteins. When dealing with self-assembling of AMPs, it is important to keep in mind that the self-assembling properties are generally associated with their secondary structure and amphiphilicity; consequently, de novo design may guarantee the self-assembling but not necessarily the antimicrobial activity.

AMPs rarely self-assemble into supramolecular structures in water solution for their cationicity, while they self-assemble into fibrillar nanostructures or helical bundles in the presence of the membrane environment, favouring an enhancement of their antimicrobial activity [88]. Instead, amphipathic AMPs can aggregate in an aqueous solution; this may have an impact on the conformational transition that usually takes place during the water-membrane interface interactions, which play a main role in the killing activity [89]. Thus, when varying the sequence of AMPs to introduce self-assembling properties, it is crucial to keep in mind how aggregation affects the interaction between the AMPs and the cell membranes. In some cases, when AMPs self-assemble into nanostructures their antibacterial efficacy is significantly enhanced [90], as is their stability which is less sensitive towards enzymatic degradation and renal clearance [91].

The antimicrobial activity can increase thanks to the conjugation of fatty acids of varying length that promote the AMP aggregation [92, 93]. Chang et al. have reported an assembled cylindrical nanostructures made from C16–V4K4 linked to an (AKKARK)_2_ heparin-binding Cardin-motif, which exhibited enhanced activity against Gram-negative bacteria above the critical micellar concentration (CMC) [94]. Similar results were reported by Beter et al. upon comparing self-assembled C_12_-VVAGKKKGRW-NH_2_ and KKKGRW-NH_2_ nanofibers with their corresponding peptide without C_12_ [95]. In contrast, Chu-Kung et al. reported that the conjugation of fatty acids of varying length to the peptide AKK (sequence: YGAAKKAA-KAAKKAAKAA), leads to a loss of antimicrobial activity [96].

Instead, self-assembling antimicrobial peptides derived from Temporin L designed by Bellavita et al. showed improved bacterial membrane interaction with consequently better antimicrobial activity and reduced cytotoxicity. In particular, the authors showed that the lipid tail of 5-carbon atoms determined a target cell specificity and a strong oligomerization in a bacterial membrane-mimicking environment and not in an aqueous solution [97]. This lipidated analogue showed the best activity against *S. aureus*, *P. aeruginosa*, and twenty resistant *K. pneumoniae* clinical isolates, as well as against some clinical *Candida albicans* species [98, 99].

A novel, versatile platform for the delivery of AMPs was developed in our laboratory [100]. As proof of concept, the peptide WMR derived from the marine AMP myxinidin [101–103] was used to decorate the surface of a fiber obtained through the arrangement of self-assembled peptide-based motifs. The multiple presentations of WMR enhanced antibiofilm activity against the Gram-negative bacterium *P. aeruginosa* and the fungus *C. albicans*. In addition, these self-assembled nanostructures also increase the stability and half-life of WMR.

The development of a supramolecular platform that acts by making holes in the cell membrane and thereby destroying the bacteria represents a very promising and alternative strategy, since bacteria are not able to develop resistance because they should completely change their membrane.

## 6. Applications for Cancer

Self-assembled nanomaterials endowed with nanorange sizes of 1–100 nm are widely used in cancer therapeutics [104]. The drug encapsulation or conjugation to a nanomaterial offers numerous advantages including targeted drug delivery, protection from proteases degradation, improved drug solubility, reduction of toxic side effects, and improved pharmacokinetic and pharmacodynamic drug properties.

Among the different nanomaterials listed above, dendrimers (such as PAMAM dendrimers) are carriers that efficiently deliver and control the anticancer drug release at the tumor site. Recently, Lewinska and co-workers functionalized the PAMAM dendrimer with lapatinib and fulvestrant drugs which act as inhibitor of EGFR and HER2 tyrosine kinases and estrogen receptor degrader, respectively [105]. The use of PAMAM dendrimer potentiated the effectiveness and cytotoxic effect of both drugs against breast cancer cells and prompted a senolytic activity against doxorubicin-induced senescent breast cancer cells. Similarly, this dendrimer was also exploited to have selective targeting and delivery of doxorubicin (Doxo) through the blood-brain barrier (BBB) and reach the brain tumor site. In this case, Ban and co-workers encapsulated Doxo in the PAMAM core, while its surface was functionalized with poly(2-methacryloyloxyethyl phosphorylcholine) (PMPC), which, being similar to the structure of acetylcholine, favours BBB permeability and brain tumor targeting [106]. This strategy has been proven to be efficacious and promising because, after the injection of the nanosystem through the tail vein, it reached the brain tumor site, released DOXO contained in its core, and induced inhibition of 81% of tumor growth.

Like dendrimers, liposomes are also a popular platform used for the targeting and controlling the release of anticancer drugs thanks to their ability to accumulate in tumor sites via the enhanced permeability and retention (EPR) effect [107]. In 1995, Doxil was the first FDA-approved liposomal formulation of Doxo [108], which had significant success in different tumor models.

In addition, due to their versatility and possibility of being functionalized, liposomes showed the ability to bypass tumor-driven resistance. For example, liposomes with encapsulated Doxo and decorated with gH625 showed a great ability to internalize in Doxo-resistant cells, inhibiting the growth of lung adenocarcinoma cancer cells [109]. Similarly, liposomes can be decorated with antibodies or specific receptor ligands to obtain a specific binding of the vector on targeted cancer cells. For instance, Iyer et al. decorated liposomes with internalizing human antibodies capable of recognizing and targeting specific epithelioid and sarcomatoid subtypes of human mesothelioma cancer [110].

Among the different types of materials, self-assembled peptide-based platforms represent one of the most recent innovations in cancer therapy [111, 112]. The design of peptide sequences drives their self-assembly and regulates the structure and function of these nanomaterials, which can respond to different environmental conditions such as pH, temperature, and ionic strength [113]. For instance, Del Genio et al. engineered self-assembled peptide-based nanofibers made of two amphiphilic peptides P1 and P2 bearing the hydrophobic (C_19_ lipid tail and hexa-alanine sequence) and hydrophilic (-GDDS- and -GKRS-) domains [114]. Both peptides were building blocks essential for the construction of nanofiber structure, while the nanofiber surface was decorated with gH625 and Doxo, as delivery peptides and anticancer drugs, respectively. Doxo was released due to the presence of a peptide sequence cleaved by the MMP-9 enzyme for on-demand delivery into triple-negative breast cancer lines [114].

Moreover, self-assembled peptide-based hydrogels also represent an important modulable and biocompatible nanomaterial used in cancer therapy. The best example is the peptide RADA16 made of repeated units of -Arg-Ala-Asp-Ala-sequence, which is able to form nanofibers with a width of 3–8 nm through electrostatic interactions [115], while at high concentrations it self-assembles, generating nanoporous hydrogels [116]. For instance, Liu and co-workers fabricated RADA16-based hydrogels with paclitaxel, obtaining a controlled drug release in MDA-MB-435S cells and an effective inhibition of tumor cell growth [117].

Self-assembled peptide nanoparticles were recently developed against hepatocellular carcinoma cells to be used for photodynamic therapy. Co-assembled nanoparticles were obtained through the combination of an aromatic short peptide with a porphyrin derivative for enhanced antitumor treatment [118].

Among the different supramolecular platforms mentioned above, polymersomes are considered a great promise due to their ability to overcome the main limitations of liposomes in drug delivery. These nanosystems own a longer half-live and a better capacity to accumulate in tumor site, releasing the drug. Significant results with polymersomes in the anticancer combined therapy were obtained by Colley et al. [119] The authors encapsulated Doxo and paclitaxel drugs into pH-sensitive PMPC- poly 2-(diisopropylamino) ethyl methacrylate (PDPA) polymersomes, which were able to penetrate rapidly into the spheroids of oral head and neck squamous cell carcinoma (HNSCC) cells, determining a significant cell damage. Interestingly, polymersomes can be customized with light-activable moieties to disassemble in response to light and obtain a controlled release of drug at tumor site in a spatiotemporal manner [120]. Hou and co-workers developed a polymersome made of poly (o-nitrobenzyl acrylate) (PNBA) and poly (N,N′-dimethylacrylamide) (PDMA), which is able to disassemble and release Doxo upon exposure to the light irradiation of 365 nm that triggers a photocleavage reaction of o-nitrobenzyl groups [121].

In this section, we briefly reported how supramolecular nanoplatforms can be designed and customized to make them able to respond at different tumor environments and obtain a targeted drug delivery. However, these nanoplatforms can also be tailored with MR imaging contrast agents for the use in cancer diagnosis.

## 7. Experimental Techniques for Biophysical Characterization

The complex process of self-assembly makes the selection of the methodology to use for biophysical characterization a key factor; actually, several experimental techniques were established to study self-assembled nanosystems with the main differences being related to the nature and size of the nanomaterial, the sensitivity, the type, and the resolution of the knowledge provided. Here, we briefly describe the most used methodologies, considering that they should always come together in a biophysical characterization of self-assembled materials. Key techniques include super-resolution fluorescence microscopy (specifically stochastic optical reconstruction microscopy (STORM)), transmission electron microscopy (TEM), atomic force microscopy (AFM), small-angle neutron scattering (SANS), circular dichroism (CD), Fourier transform infrared spectroscopy (FTIR), and nuclear magnetic resonance (NMR)).

The use of TEM, AFM, and optical microscopy provides evidence about morphologies and geometric parameters, such as size and diameter. Spectroscopic methods such as CD, NMR, and FTIR are suitable to determine the secondary structures of self-assembled materials. Moreover, scattering techniques such as SANS are broadly used, but elucidation of results might be difficult.

One of the most used methodologies to analyse the establishment of aggregated structures is fluorescence spectroscopy. Fluorophores such as Nile Red and Thioflavin T can be exploited to investigate their diverse environment when in aqueous solutions and when in the hydrophobic core of the self-assembled structure. In addition, labelling of peptides and/or lipids with a fluorescent tag may be used to analyse the kinetics of self-assembly. Tryptophan (Trp) residues are present or are added to peptides for their intrinsic fluorescence, which is very sensitive to the polarity of the environment. Furthermore, supplementary data may be acquired using polar Trp quenchers such as iodide or acrylamide, while brominated or methyl-coumarin-labelled phosphocholines can be used to quench the Trp in hydrophobic environments.

Regarding the STORM technique, self-assembling nanosystems are firstly conjugated with fluorescent dyes, which are then activated by lasers to induce a blinking state [122]. The position of dye molecules is determined with high precision, and super-resolution images can be obtained by combining all the fluorescent dyes. Thus, labelling with different dyes allows STORM to distinguish between molecules of the same kind with minimum invasive sample preparation.

AFM consents to determining the density of self-assembled structures which usually appear to be higher than that determined by STORM; in fact, while STORM images are acquired in bulk solution, AFM images are recorded after applying the drying process to cause artificial condensation onto the substrate surface. Clearly, AFM does not provide any chemical information of the analysed system.

TEM is chosen for the nanometric investigation of the structure and morphology of self-assembled structures [123]. The strongest advantage of this technique is the ability to withdraw detailed information on individual species present inside a population. Nonetheless, since conclusions are based on the observation of a limited number of objects, TEM should always be reinforced by adding data obtained from other techniques such as scattering and spectroscopy.

SANS is a nondestructive technique based on the intensity of a neutron beam scattered from a solution [124]. First, the structural features can be derived from the calculation of the theoretical scattering profile achieved from analytic models which is then fitted to the measured data. SANS is strongly complementary to TEM and AFM imaging techniques in terms of inner diameter and wall thickness determination.

In case of peptides, the secondary structure as well as the stability of the self-assembled nanosystem to dilution, different pH values, and different ionic strength can easily be obtained from CD. Furthermore, FTIR spectroscopy can be used to study self-assembly upon drying nanostructures, generating great insight into the dynamics of these processes. The molecular information gained by FTIR allows the determination of the structural and conformational changes of functional groups [125].

NMR is generally used to complement CD to determine the secondary structure of self-assembled aggregates. Solution measurements are limited to the mobility of molecules because the assembly reduces the intensity and shifts the frequency, causing difficulties in interpreting the spectra [126]; while solid-state NMR (that are often formed from isotope-labelled peptides such as 2H, 13C, 15N, and 31P to allow specific labelling) represents an effective alternative [127].

In conclusion, despite the huge assortment of experimental techniques that are available, the full characterization of interactions in self-assembled nanosystems is still a complex process which needs the use of several complementary experimental techniques.

## 8. Conclusions

**Figure 5:**
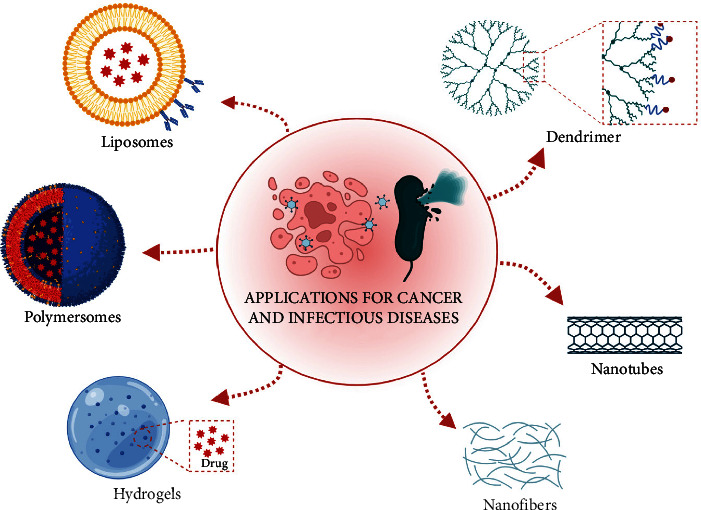
The overview of supramolecular nanomaterials used in anticancer and antimicrobial fields. This figure was created with https://BioRender.com.

In summary, we presented in detail supramolecular materials exhibiting wide applications in different fields ranging from drug delivery to biosensing, bioimaging, and tissue engineering. Self-assembly is a phenomenon controlled by internal and external molecular interactions such as hydrogen bonding, hydrophobic, and electrostatic interactions. These interactions with other factors provide the way for forming the kind of nanostructure such as fibers, nanotubes, vesicles, and micelles. The most common nanomaterials, such as liposomes, micelles, and dendrimers, are extensively used as platforms for achieving targeted drug delivery in anticancer and antimicrobial fields (see Figure 5). The possibility to polyfunctionalize them with delivery and targeting moieties and drugs depending on the kind of pathology has allowed for significant results in the biomedical field. However, herein, we also demonstrated how self-assembled peptide-based nanoplatforms have recently acquired great attention thanks to their biocompatibility, biodegradability, and versatility. Although these nanomaterials are still little known, they exhibit efficacy, good tumor environment response, and the capacity to control the drug release, yielding promising results both in the anticancer and antimicrobial fields. Overall, we believe that supramolecular nanomaterials can overcome issues related to drugs and their side effects and can be used both in diagnosis and treatment, being designed and engineered *ad hoc* as a function of their use.

## Data Availability

The data used to support this study are included within the article.
